# Fractured T Tube Fragment in Common Bile Duct during Retrieval: An Unforeseen Mishap

**DOI:** 10.1055/s-0041-1735643

**Published:** 2021-09-14

**Authors:** Deepak Rajput, Itish Patnaik, Sruthi Shasheendran, Beeram K. Prasanna Kumar, Amit Gupta

**Affiliations:** 1 Department of General Surgery, All India Institute of Medical Sciences, Rishikesh, Dehradun, India; 2 Department of Medical Gastroenterology, All India Institute of Medical Sciences, Rishikesh, Dehradun, India

**Keywords:** choledocholithiasis, CBD exploration, T tube fragment retrieval, ERCP

## Abstract

Common bile duct (CBD) exploration by surgical method—open or laparoscopic, traditionally involved using a T tube to take care of postoperative intraluminal pressure and edema. The complications of T tube include bile leak after removal, formation of biliary fistula, excoriation of the skin, dehydration, saline depletion, retained T tube fragment, CBD obstruction, cholangitis, pancreatitis, and duodenal erosion. Here, we report a case of retained T tube fragment after an attempted removal in an operated case of choledocholithiasis, which was managed by endoscopic retrograde cholangiopancreatography and balloon catheter removal of the remnant.

The rationale of using a T tube after choledochotomy includes controlled biliary drainage, prevention of biliary leak, promote healing of choledochotomy, and providing a tract for radiological evaluation of biliary tree patency. Morbidity occurring with the T tube in situ includes fluid and electrolyte disturbances, sepsis, premature dislodgement, and bile leakage. In addition, bile leakage after removal can lead to biliary ascites, biloma, or bile peritonitis. Other complications include bile duct trauma, prolonged fistula, and stricture formation.

## Case Report

A 27-year-old woman, without comorbidities, presented to the emergency with complaints of right upper abdominal pain and yellowish discoloration of urine, eyes of 1-month duration, and associated fever, itching of 2 weeks duration. There was no history of loss of weight or appetite. There were no cardiorespiratory, urinary, or neurological symptoms, and past history (similar complaints or previous surgery) was unremarkable. Laboratory workup revealed leucocyte count: 16 × 10 ^9^ /L, conjugated hyperbilirubinemia with total and direct bilirubin 342 and 198.36 µmol/L, respectively, and alkaline phosphatase value of 3,010 IU/L. Hepatobiliary ultrasound scan showed an enlarged liver ∼17.8 cm, moderate dilatation of intrahepatic biliary radicles (IHBRs), and dilated common bile duct (CBD) ∼15 mm with smooth tapering at the lower end.

**Fig. 1:**
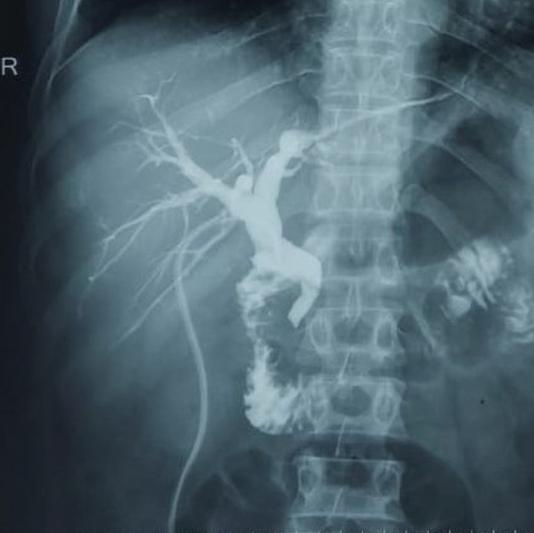
T tube cholangiogram showing patent biliary tree.

A provisional diagnosis of obstructive jaundice due to choledocholithiasis with cholangitis was made. After starting intravenous antibiotics, the patient underwent an endoscopic retrograde cholangiopancreatography (ERCP), but it was unsuccessful due to pancreatic duct cannulation. Magnetic resonance cholangiopancreatography (MRCP) revealed a 12 × 16.7 mm filling defect in distal CBD (∼14 mm) with bilobar IHBR dilatation. An option of percutaneous transhepatic biliary drainage was also offered to the patient. Still, an assessment of the procedure on the grounds of cost, benefits, and risks led to her refusal. The patient had remission of febrile episodes over the next 72 hours, hence an upfront exploratory laparotomy with cholecystectomy and CBD exploration with 12 Fr T tube (Kehr's “T” Tube; Romsons Scientific & Surgical Industries Pvt. Ltd.) insertion was performed after taking informed consent. The stone could be located with difficulty after repeated instrumentation, impacted at the junction of the cystic duct (low and posterolateral insertion), and CBD with mucosa encroaching over it like a flap. After the stone extraction, there was a free passage of the irrigating fluid and a 6-Fr infant feeding tube into the duodenum. The postoperative period was uneventful; a T tube cholangiogram ( Fig. 1 ) done on day 10 showed no biliary leak; hence, the patient was discharged on a postoperative day 15 with a blocked T tube in situ.

**Fig. 2:**
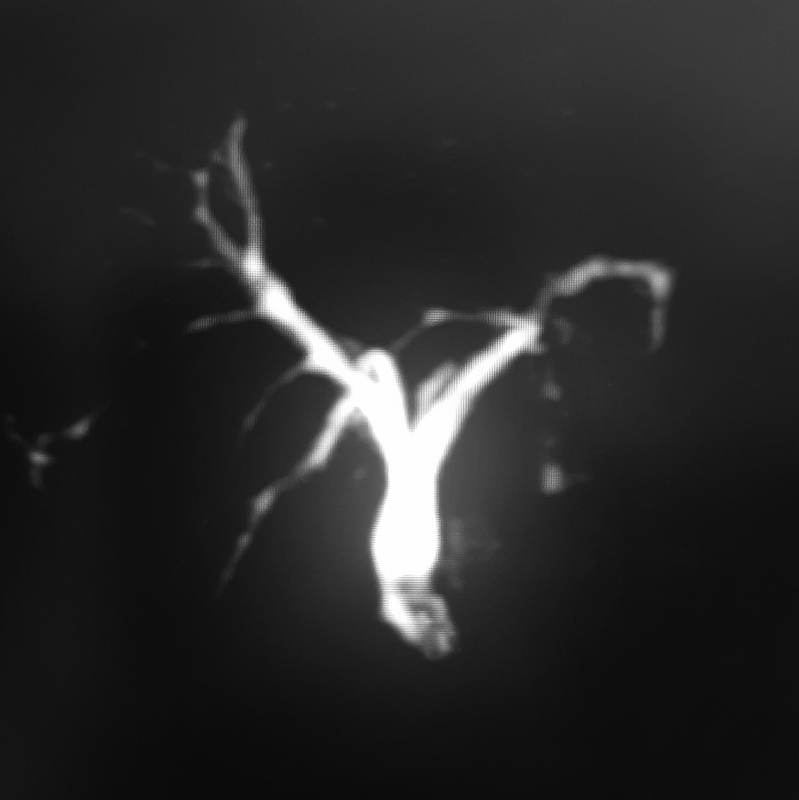
Magnetic resonance cholangiopancreatography film showing retained T tube fragment in common bile duct lumen.

**Fig. 3:**
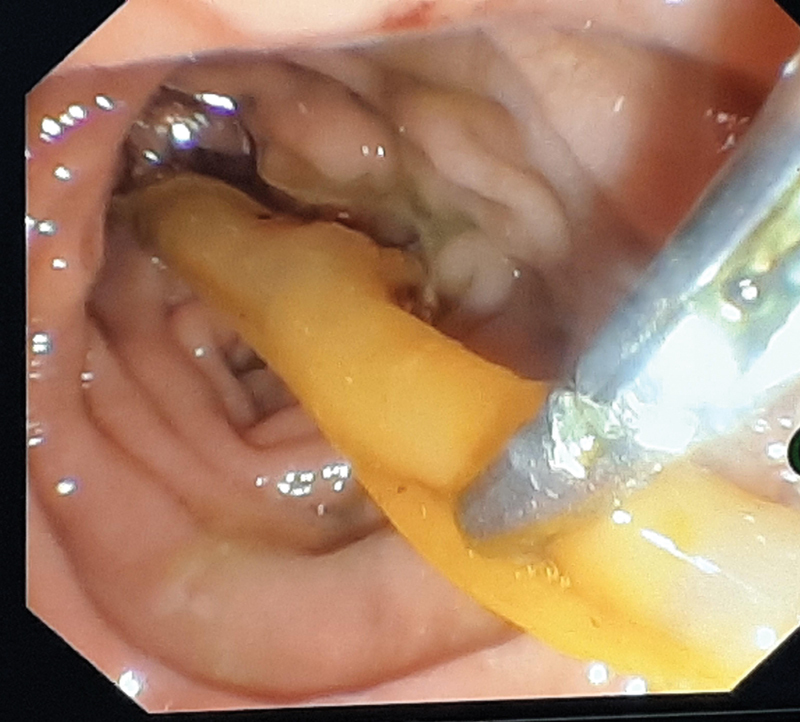
Endoscopic retrieval of T tube fragment.

**Fig. 4:**
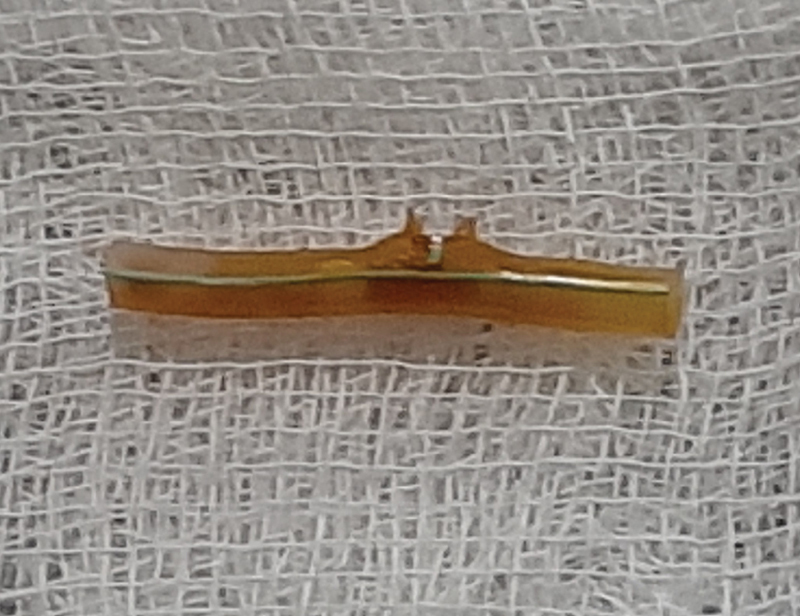
Retrieved T tube fragment in entirety.

On follow-up at 3 weeks, the patient was doing well, and hence, T tube removal was planned. However, it got fractured at the junction between the short and long limbs on attempted removal, and only the long segment of the T tube could be retrieved. The patient was admitted and kept under close monitoring. A plain X-ray abdomen was done, which showed radiopaque linear intensity over the right paravertebral location suggestive of a retained T tube fragment. MRCP showed dilated CBD ∼9.3 mm with a T tube remnant approximately 2 cm length, in mid and distal CBDs ( Fig. 2 ). ERCP, done this time by a different endoscopist, showed a radiopaque structure in the mid and distal CBDs, retrieved by balloon catheter postsphincterotomy followed by the placement of a bile duct stent ( Fig. 3 ). T tube fragment was inspected for its entirety ( Fig. 4 ). No adverse events were reported at 2 weeks to follow, and the CBD stent was removed without any subsequent complications.

## Discussion

There are several approaches for biliary duct stone clearance in the current era, both surgical and nonsurgical. The surgical approach can be open or laparoscopic CBD exploration, and the nonsurgical procedure includes ERCP. Over the years, minimally invasive techniques have gained preponderance over open methods. Guidelines have been formulated to aid the surgeon in managing CBD stones in several clinical scenarios. ERCP has been advised to treat CBD stones in patients of postcholecystectomy status, severe cholangitis, or pancreatitis. For patients presenting with gall stone disease and CBD stones, a single-stage procedure is preferable. Laparoscopic cholecystectomy combined with CBD exploration (LCBDE) is equivalent to laparoscopic cholecystectomy with intraoperative ERCP in terms of overall morbidity and mortality. LCBDE with either primary repair of choledochotomy alone or simultaneous T tube drainage has been widely practiced. CBD exploration with primary bile duct closure was found to have a shorter operating time, shorter duration of hospital stay, and lower incidence of postoperative biliary peritonitis when compared with CBD exploration followed by T tube drainage. 1

The traditional management of choledochotomy involved closing the incision over a T tube. The complications of T tube include bile leak after removal, formation of biliary fistula, bacteremia, wound sepsis, retained T tube fragment, CBD obstruction, cholangitis, pancreatitis, and duodenal erosion. Of these, the common complications include bile leak, peritonitis, biliary fistula, and postoperative biliary stenosis. The most frequent is bile leak reported in 1 to 19% of cases. 2 Retained T tube fragment is an infrequent complication of T tube removal. 3 It has been reported to cause delayed cholangitis. 4 Retained T tube fragment causing bile duct stone formation has also been reported. 5 The retained fragment can be removed endoscopically and, if not feasible, may require surgical extraction. In our case, as the stone was impacted and could be located only after repeated manipulation of the bile duct owing to the anatomical variation of the insertion of cystic duct, we closed the choledochotomy incision over a T tube, anticipating impaired healing.

A literature review shows only a few reported cases of a T tube fragment retained in the CBD. Of these, the timing of presentation varied from the immediate postoperative period to several years. In six cases, patients presented with cholangitis and had a retained fragment for 2 to 36 years which was removed by ERCP and sphincterotomy in five instances and surgically in one case. 6 7 8 9 10 Nearly four cases recognized the fracture of the T tube at the time of removal, and hence, immediate retrieval was done. 11 12 13 14 The retained T tube fragments were retrieved by ERCP, percutaneous balloon catheters, and surgically. ERCP and Dormia basket removal of T tube fragments have been successfully achieved as early as the 1990s. 15

We report a rare case of retained T tube fragment during its removal in the hospital managed endoscopically. Our case illustrates that the T tube should be removed under the supervision of the treating doctor and inspected for its entirety postretrieval. Also, the patient should be shown a fashioned T tube so that a retained fragment does not go unnoticed if the tube gets accidentally pulled out at home.

## Conclusion

**Fig. 5:**
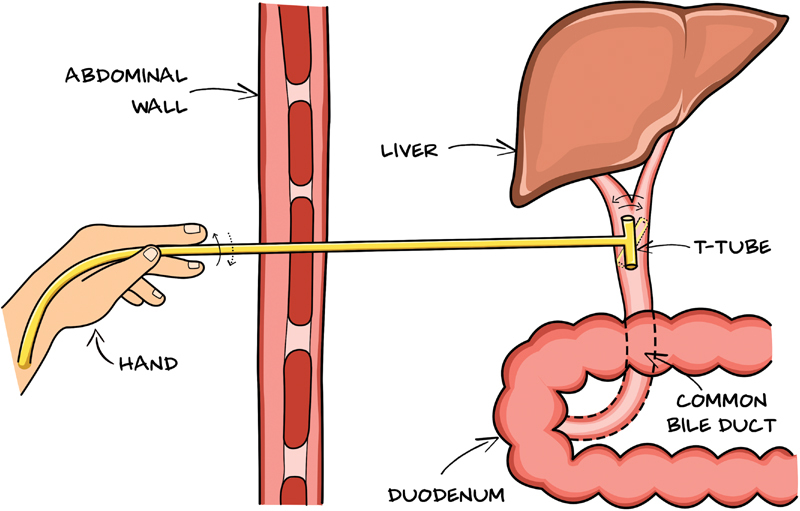
Illustration showing the roll of T tube before pulling out.

The T tube should be initially rolled between the thumb and the index fingertips and subsequently flushed with normal saline if it does not come out quickly ( Fig. 5 ). Any retained T tube fragment should be retrieved immediately to avoid early and late complications. As there is no standard method to retrieve retained T tube fragments, endoscopic removal, even though technically challenging, is safe and effective and should be attempted first to avoid major second surgery.
